# Optimizing and Characterizing Geopolymers from Ternary Blend of Philippine Coal Fly Ash, Coal Bottom Ash and Rice Hull Ash

**DOI:** 10.3390/ma9070580

**Published:** 2016-07-15

**Authors:** Martin Ernesto Kalaw, Alvin Culaba, Hirofumi Hinode, Winarto Kurniawan, Susan Gallardo, Michael Angelo Promentilla

**Affiliations:** 1 Mechanical Engineering Department, De La Salle University, Manila 1004, Philippines; alvin.culaba@dlsu.edu.ph; 2 International Development Engineering, Tokyo Institute of Technology, Tokyo 152-8550, Japan; hinode.h.aa@m.titech.ac.jp (H.H.); kurniawan.w.ab@m.titech.ac.jp (W.K.); 3 Chemical Engineering Department, De La Salle University, Manila 1004, Philippines; susan.gallardo@dlsu.edu.ph (S.G.); michael.promentilla@dlsu.edu.ph (M.A.P.)

**Keywords:** alkali activation, bottom ash, fly ash, geopolymer, rice hull ash, sustainability, waste utilization

## Abstract

Geopolymers are inorganic polymers formed from the alkaline activation of amorphous alumino-silicate materials resulting in a three-dimensional polymeric network. As a class of materials, it is seen to have the potential of replacing ordinary Portland cement (OPC), which for more than a hundred years has been the binder of choice for structural and building applications. Geopolymers have emerged as a sustainable option vis-à-vis OPC for three reasons: (1) their technical properties are comparable if not better; (2) they can be produced from industrial wastes; and (3) within reasonable constraints, their production requires less energy and emits significantly less CO_2_. In the Philippines, the use of coal ash, as the alumina- and silica- rich geopolymer precursor, is being considered as one of the options for sustainable management of coal ash generation from coal-fired power plants. However, most geopolymer mixes (and the prevalent blended OPC) use only coal fly ash. The coal bottom ash, having very few applications, remains relegated to dumpsites. Rice hull ash, from biomass-fired plants, is another silica-rich geopolymer precursor material from another significantly produced waste in the country with only minimal utilization. In this study, geopolymer samples were formed from the mixture of coal ash, using both coal fly ash (CFA) and coal bottom ash (CBA), and rice hull ash (RHA). The raw materials used for the geopolymerization process were characterized using X-ray fluorescence spectroscopy (XRF) for elemental and X-ray diffraction (XRD) for mineralogical composition. The raw materials’ thermal stability and loss on ignition (LOI) were determined using thermogravimetric analysis (TGA) and reactivity via dissolution tests and inductively-coupled plasma mass spectrometry (ICP) analysis. The mechanical, thermal and microstructural properties of the geopolymers formed were analyzed using compression tests, Fourier transform infra-red spectroscopy (FTIR), scanning electron microscopy (SEM) and thermogravimetric analysis (TGA). Using a Scheffé-based mixture design, targeting applications with low thermal conductivity, light weight and moderate strength and allowing for a maximum of five percent by mass of rice hull ash in consideration of the waste utilization of all three components, it has been determined that an 85-10-5 by weight ratio of CFA-CBA-RHA activated with 80-20 by mass ratio of 12 M NaOH and sodium silicate (55% H_2_O, modulus = 3) produced geopolymers with a compressive strength of 18.5 MPa, a volumetric weight of 1660 kg/m^3^ and a thermal conductivity of 0.457 W/m-°C at 28-day curing when pre-cured at 80 °C for 24 h. For this study, the estimates of embodied energy and CO_2_ were all below 1.7 MJ/kg and 0.12 kg CO_2_/kg, respectively.

## 1. Introduction

Since 2012, the annual coal consumption in the Philippines was 18 million metric tons (MMT), which has increased by an average of 10% in the previous five years [1]. This, together with the more than 1.4 MMT of annual coal ash generation [2], is expected to increase significantly as more than twenty coal fired power plants will be built in the coming years [3,4]. Clean coal combustion technology may address the emissions issues; however, coal ash disposal and management problems due to increasing coal ash generation and accumulation are threatening concerns. As generation quantities are large, large-scale utilizations are also necessitated to alleviate disposal issues. Among these utilization strategies, the one with the highest potential impact is in the use of coal ashes as the main constituent of alternative binders for building and structural applications [5]. More and more coal fly ash are being used as a component in blended OPC (up to 55% in CEM IV, European standards EN 197-1) and high volume fly ash (HVFA) concrete (up to 70%) as supplementary cementitious materials (SCM) [6]. This helps in the reduction of the use of natural resources, like mined limestone and sand, as well as the lessening of carbon dioxide emissions compared to pure OPC production [7,8]. However, the development of geopolymerization processes opens opportunities for alternative binders entirely free of OPC [9,10,11] to be produced from alumina- and silica-rich industrial waste materials, such as coal ash. Potentially, this may come with the additional environmental benefit of as much as an 80% reduction in CO_2_ emissions [12].

Many geopolymer formulations have been investigated and shown to have comparable if not better technical properties than OPC [7]; however, different precursor materials, new blending components and processing conditions affect the geopolymer reactions and present different mechanical properties and microstructures [5]. It has also been projected that geopolymers can be formulated to address specific niche applications [5,7]. It follows that the processing requirements can be managed to produce the desired geopolymer characteristics in the most practicable means possible [5].

Fly ash-based geopolymers have been investigated with only coal fly ash (CFA) as the alumino-silicate source or with blending components as metakaolin [13], fibers such as carbon fibers [14] and polypropylene [15] and slag [16,17], among others. The reported compressive strength reached more than 60 MPa [18,19] with each blending component affecting such properties as electrical conductivity, modulus of elasticity and, in general, compressive strength. Bottom ash has also been used as a primary component in geopolymerization [20,21,22,23,24,25] and also as a combining material for fly ash-based geopolymers [26]. The compressive strength results obtained by Antunes Boca Santa et al. (2013) with bottom ash as the primary component range from 10–25 MPa [20]. Rice hull ash, a known source of amorphous silica, has been used in geopolymers as a primary source of silica in ternary mixtures with red mud and diatomaceous earth [27] and as a supplementary source in fly ash-based geopolymers [28,29].

In the Philippines, five major coal-fired power plants alone produce about 1.4 MMT of coal ash per year [2] with a very low percentage of the utilization of fly ash and almost nil for bottom ash. As its generation increases, coal ash requires more and more landfill area. As specific cases, power plants in the cities of Naga and Toledo and even a new plant in the Visayas region have projected remaining landfill capacities of less than a decade [2,30]. However, more than the issue of increasing need for landfill area, coal ash disposal poses the threat of the contamination of surface waters and groundwater from its heavy metal and mineral contents [2]. Furthermore, being an agricultural country whose staple is rice with 19 MMT of paddy rice production in 2015 [31], rice hull as a waste stream is also produced in large quantities that far outweigh its utilization. This also results in disposal problems.

In this study, to explore the potential of the sustainable waste utilization of coal ashes and rice hulls, the properties of geopolymers from the mixture of coal fly ash (CFA) and coal bottom ash (CBA) from the combustion of coal in coal-fired power plants and rice hull ash (RHA) from the use of rice hulls as biomass fuel were evaluated. The raw materials, CFA, CBA and RHA, were characterized using X-ray fluorescence (XRF) spectroscopy, Fourier transform infra-red (FTIR) spectroscopy, X-ray diffraction (XRD), thermogravimetric analysis (TGA) and scanning electron microscopy (SEM). Dissolution tests and inductively-coupled plasma (ICP) mass spectrometry on the alkali solvent were also done to evaluate the reactivity of the raw materials. A mixture design using a Scheffé-based model was used to determine the mix proportions and to obtain the optimum mix conditions of a CFA-CBA-RHA ternary mix geopolymer for a target application requiring low thermal conductivity, light weight and medium strength structural material. The mechanical, thermal and microstructural properties of the geopolymer samples formed were also evaluated using compression tests, FTIR, SEM and TGA. Finally, to assess the environmental impact, embodied energy and embodied CO_2_ were estimated for the process.

## 2. Materials and Methodology

### 2.1. Sources of Raw Materials

The rice hull ash (RHA) was obtained from a small rice hull fired power plant serving an industrial plant south of Manila. The coal fly ash (CFA) and coal bottom ash (CBA) were obtained from a coal fired power plant from the southeast region of Luzon, Philippines. The alkali activator was prepared from locally-available reagents at an 80-20 by mass ratio of 12 M NaOH (sodium hydroxide) solution and Na_2_SiO_3_ (sodium silicate or waterglass) solution (55% water, modulus = 3). This mass ratio was based on preliminary experiments done for the local raw materials to achieve a geopolymer mix with considerable compressive strength and good workability [32]. Mixtures of NaOH, among the alkali hydroxides, and waterglass, among alkali silicates, are the most commonly-used alkali activator mixtures for geopolymerization [33,34]. In a study [35] using 10 M NaOH and waterglass solution (66.1% water, modulus = 3.2), a higher amount of waterglass solution, 0.5–1.0 by mass ratio to NaOH, produced better strength. However, Fernandez-Jimenez et al. [36] using a mixture of 85% 12.5 M NaOH and 15% waterglass solution were able to produce alkali-activated fly ash concrete with compressive strength near 60 MPa for 15-cm cube specimens thermally cured at 85 °C for 20 h and then stored at room conditions. In this study, the choice of using more of NaOH in the alkali activator solution is of practical significance since NaOH is a cheaper and widely available reagent.

### 2.2. Characterization of Raw Materials

XRD and XRF were used to determine the mineralogical characteristics and elemental analysis of the ashes, respectively. XRD was conducted via a Multiflex Rigaku Automated Powder X-ray Diffractometer (Rigaku Corp., Tokyo, Japan) (λ_Cu Kα_ = 1.54 Å) equipped with a goniometer having a diameter of 185 mm using a measuring angle from 5°–60°. XRF was done using a Sequential X-ray Fluorescence Spectrometer ARL ADVANT’X IntelliPower Series (Thermo Scientific, Waltham, MA, USA). SEM was used to examine the microstructure, whereas thermal analysis via TGA was also done on both raw materials and geopolymers. SEM used the JEOL JSM-5310 SEM (Jeol Ltd., Tokyo, Japan) with a thermionic tungsten filament electron source, and TGA used the Rigaku Thermo Plus Model TG 8120 SEM (Rigaku Corp., Tokyo, Japan) using alumina as the reference at a 10 °C/min heating rate from ambient temperature to 800 °C. The FTIR was done using a Jasco FT/IR-6100FV Fourier Transform Infrared Spectrometer SEM (Jasco Intl., Tokyo, Japan) on samples mixed with KBr pellets and pulverized in a mortar and pestle.

### 2.3. Dissolution Tests

The method used in the dissolution tests was adapted from the procedure outlined in Chen-Tan and van Riessen [37]. The dissolution experiments were conducted at room temperature. Two-point-five grams of CFA, 2.5 g of CBA (ground and sieved at 250 µm) and 2.0 g of RHA samples each were dissolved in 50 mL of 10 M NaOH solution. For RHA only, 2.0 g of RHA were used because of the higher sample volume. The solutions were stirred continuously for 24 h using magnetic stirrers. The solid residues were filtered out using 0.2-mm filter papers. The filter papers with the residues were dried at 105 °C until the weight no longer changed. The weight of the filter paper used was subtracted to obtain the mass of undissolved sample and then compared to the initial sample mass used to determine the percent of dissolution.

After the dissolution tests, a portion of the filtrate was diluted to ten parts water and then passed through the inductively-coupled plasma mass spectrometry (ICP) analyzer to determine the elemental components dissolved in the NaOH solution.

### 2.4. Pre-Treatment of Raw Materials and the Preparation of Geopolymer Specimens

**Figure 1:**
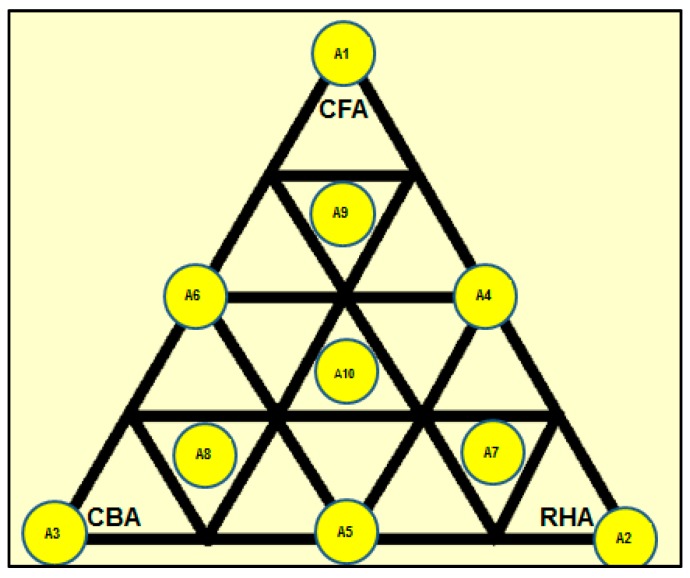
Mix proportions used.

As minimal raw material processing was considered, only the CBA was ground and passed through a 250-μm sieve size. The RHA, being very soft and brittle, was ground, but was no longer sieved. The materials were mixed according to mix proportions determined using the Scheffé mixture design, as shown in Table 1 and Figure 1. The alkali activator was added at 20% by weight of dry materials and mixed for 20 min using a motorized 2-L capacity laboratory mixer. Additional water was slowly added to maintain the workability of the slurry formed. Then, the slurry was poured into 50-mm cubical molds and 50-mm × 50-mm × 100-mm molds. The molds were then repeatedly tapped onto the work tables to manually vibrate the samples to release air bubbles. For the mixture design, 3 replicates of each specimen were cured for 28 days at room conditions.

The specimens were allowed to set in the molds for 24 h, then de-molded and then cured for 27 more days at ambient conditions. After the 28-day curing period, the volumetric weight (average density) was determined for each of the 50-mm cubical specimens. These specimens were then tested for compressive strength using a Digimax Plus automatic Uniframe compression tester (Controls s.r.l., Milan, Italy). The thermal conductivity was measured from the 50-mm × 50-mm × 100-mm specimens using the QTM-500 thermal conductivity meter (KEM, Kyoto, Japan).

### 2.5. Multiple Response Surface Optimization via Desirability Functions

Using multiple response surface optimization via desirability functions, the optimal mix formulation of the ternary blend was determined by maximizing the overall desirability of the geopolymer product [27,38,39,40,41]. The overall desirability *D* given in Equation (1) refers to the weighted geometric mean of the individual desirability values, *d_i_*(*Y_i_*), each of which ranges from 0 to 1 [27,41]. These individual desirability functions are dependent on the values of the response variables *Y_i_* (e.g., volumetric weight, compressive strength and thermal conductivity) which are in turn functions of the input variables (mass fractions F, B, R of CFA, CBA and RHA, respectively), as given by Equation (2).

(1) $$D=\prod_{i=1}^{k}d_{i}{\left(Y_{i}\right)}^{w_{i}}$$

where:

(2) $$Y_{i}=f(F,B,R)$$

*w_i_* = importance weight of the response variables, such that $\sum_{\text{i}=1}^{k}w_{i}=1$, *k* = the number of response variables.

The individual desirability function, *d_i_*(*Y_i_*), is determined from Equation (3) if the response *Y_i_* is set to approach a target value *T_i_*. *L_i_* and *U_i_* are the lowest and highest values, respectively of the response variable considered.

(3) $$d_{i}(Y_{i})=\{\begin{array}{cc} 0 & Y_{i}<L_{i}\\ {\left(\frac{Y_{i}-L_{i}}{T_{i}-L_{i}}\right)}^{s} & L_{i}\leq Y_{i}\leq T_{i}\\ {\left(\frac{Y_{i}-U_{i}}{T_{i}-U_{i}}\right)}^{t} & T_{i}\leq Y_{i}\leq U_{i}\\ 0 & Y_{i}>U_{i} \end{array}$$

The *d_i_*(*Y_i_*) is determined from Equation (4) if the response is to be maximized. Here, *T_i_* is the value taken as large enough, and *L_i_* is the lowest measured value for the response considered.

(4) $$d_{i}(Y_{i})=\{\begin{array}{cc} 0 & Y_{i}<L_{i}\\ {\left(\frac{Y_{i}-L_{i}}{T_{i}-L_{i}}\right)}^{s} & L_{i}\leq Y_{i}\leq T_{i}\\ 1 & Y_{i}>T_{i} \end{array}$$

Additionally, *d_i_*(*Y_i_*) is determined from Equation (5) if the response is to be minimized. In this case, *T_i_* is the value taken as small enough, and *U_i_* is the highest measured value for the response considered.

(5) $$d_{i}(Y_{i})=\{\begin{array}{cc} 1 & Y_{i}<T_{i}\\ {\left(\frac{Y_{i}-U_{i}}{T_{i}-U_{i}}\right)}^{t} & T_{i}\leq Y_{i}\leq U_{i}\\ 0 & Y_{i}>U_{i} \end{array}$$

The parameters *s* and *t* determine how the desirability function, *d_i_*(*Y_i_*), behaves. For *s* = *t* = 1, *d_i_*(*Y_i_*) increases towards *T_i_* linearly; for *s* < 1, *t* < 1, *d_i_*(*Y_i_*) is convex; and for *s* > 1, *t* > 1, *d_i_*(*Y_i_*) is concave [40].

For this study, assuming the response variables are equally important, the optimization model is:

Maximize:

(6) $$D=\sqrt[{k}]{d_{1}(Y_{1})\cdot d_{2}(Y_{2})\cdot \cdot \cdot d_{k}(Y_{k})}$$

where $Y_{i}=f(F,B,R)$ for *i* = 1 to *k*.

Subject to:

(7) 0≤F≤1

(8) 0≤B≤1

(9) 0≤R≤1

(10) F+B+R=1

Equations (7)–(9) indicate the limiting values of the dry mass fractions of the individual components, and Equation (10) indicates that only the three components comprise the entire mixture.

For example, for the target attributes of the geopolymer product, one set of conditions was to maximize the compressive strength, using Equation (3) and to minimize the volumetric weight and thermal conductivity using Equation (4). In addition, another set of conditions was set to a minimum compressive strength of 11.7 MPa and a maximum volumetric weight of 1680 kg/m^3^ to meet the performance specifications of ASTM C90-14 for moderately-loaded, lightweight load bearing concrete [42] and ASTM C0109/C0109M for the Standard Test Method for Compressive Strength of Hydraulic Cement Mortars (using 50-mm cube specimens) [43].

After the optimal mixes were determined, additional geopolymer specimens were prepared using the optimal mix ratio. Because of the observed bigger pores in the first set of specimens, which can be attributed to air bubbles, longer and more controlled manual vibration was done on the optimal mix specimens. To check the effect of elevated temperature pre-curing [26,36], one set of specimens was set in the mold for 24 h, then de-molded and then pre-cured at 80 °C in a convection oven for 24 h with the remainder of the 28-day curing period done at ambient temperature.

### 2.6. Evaluation of Embodied Energy and Embodied CO_2_ of the Optimized Geopolymer Mix

Finally, to check on environmental impact, embodied energy and embodied CO_2_ were estimated for the geopolymer produced and compared to that of OPC. Embodied energy and embodied CO_2_ of a product, in this case geopolymer cement or OPC, represent all of the associated energy needed and CO_2_ generated to produce the product from its raw materials to material processing up to the finished product until the end of its useful life, referred to as “cradle to grave” [44,45,46]. However, the current common practice is to specify these quantities as from “cradle to gate” (until the products leave the factory gate). Hammond and Jones [44] developed the Inventory of Carbon and Energy (ICE), a list of energy intensities of a variety of building materials and their respective carbon implications, calculated using the “cradle to gate” approach, which considers all energy consumption from upstream stages, such as raw material extraction, to the final stage as a finished product. From this database, OPC has an embodied energy of 4.6 MJ/kg and an embodied CO_2_ of 0.83 kg CO_2_/kg. For fly ash blended OPC, the embodied energy is 3.52 MJ/kg with 25% replacement and 2.43 MJ/kg with 50% replacement. The corresponding embodied CO_2_ is 0.62 kg CO_2_/kg and 0.42 kg CO_2_/kg for 25% and 50% OPC replacement with fly ash, respectively.

Using the same “cradle to gate” approach, the system boundary considered for this study was from the raw materials and reagents used until the geopolymers are cured. The coal ashes, fly ash and bottom ash, and rice hull ash were considered to enter the system boundary with no embodied impacts, as the impacts were placed on the products (electricity for the coal and the rice hulls used as fuel for electricity generation) [47].

## 3. Results and Discussion

### 3.1. Characterization of Raw Materials

Table 2 shows the composition of the raw materials via XRF. As seen from the table, CFA and CBA have significant amounts of alumina and silica, which make them good precursors for geopolymerization. CBA has higher amounts of CaO and Fe_2_O_3_. Both CFA and CBA have the same SiO_2_/Al_2_O_3_ ratios of 5.2, which is within the range of values (3.3–5.9) for good geopolymerization [48,49]. However, it is noted that the RHA has a high loss on ignition (LOI) of 28.6%. The low values of alkali metals as seen in the K_2_O/Al_2_O_3_ and K_2_O/SiO_2_ ratios are supplemented by the Na in the NaOH/waterglass activator solution.

Geopolymerization is dependent on the reactivity of the materials used. Reactivity is indirectly measured via the dissolution of the amorphous alumina and silica in the alkaline activator solution [37,50]. The dissolution tests were conducted with 10 M NaOH as the solvent using a simplified form of the methodology used by Chen-Tan and van Riessen [37]. For this reactivity characterization, one set of dissolution tests at room conditions was done. The results are shown in Table 3.

After the dissolution tests, part of the filtrate was diluted to ten parts water and then analyzed using ICP to determine the dissolved components in the solution. The results are shown in Table 4.

From the results of the dissolution tests, CFA has the highest dissolution rate at 30.7%, followed by RHA at 24.0%, and CBA has the least dissolution at 14.1%. In the ICP analyses, almost all components have higher amounts dissolved from CFA than CBA. This validates the observation that the CFA is more reactive than the CBA. The reactivity test results can also be attributed to the much smaller size of CFA than CBA (<250 µm) and the degree of amorphousness, as can be seen from the XRD and SEM analyses below.

**Figure 2:**
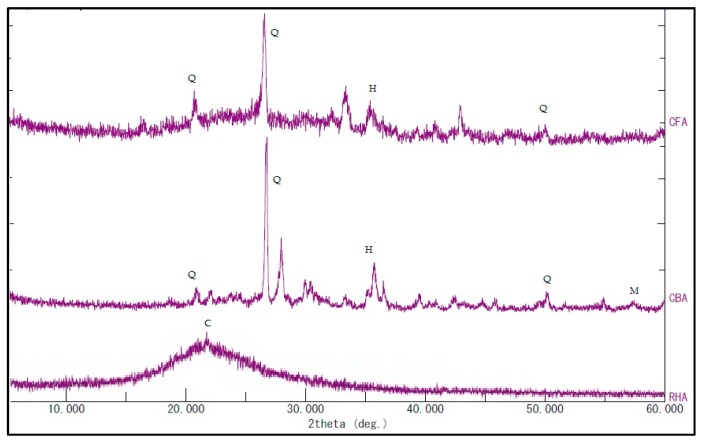
XRD patterns of the raw materials. C, cristobalite-SiO_2_; Q, quartz-SiO_2_; H, hematite; M, magnetite.

X-ray diffractograms of the raw materials, as shown in Figure 2, were taken to determine the crystalline phases present in the raw materials. The phases identified were cristobalite-SiO_2_ for RHA and quartz-SiO_2_, hematite and magnetite for CFA and CBA.

The wide hump in the RHA diffractogram between 2θ of 15° and 25° indicates that the RHA is generally amorphous silica. The wider hump under the quartz peak of CFA compared to CBA also indicates that the CFA has more amorphous silica than CBA [26].

**Figure 3:**
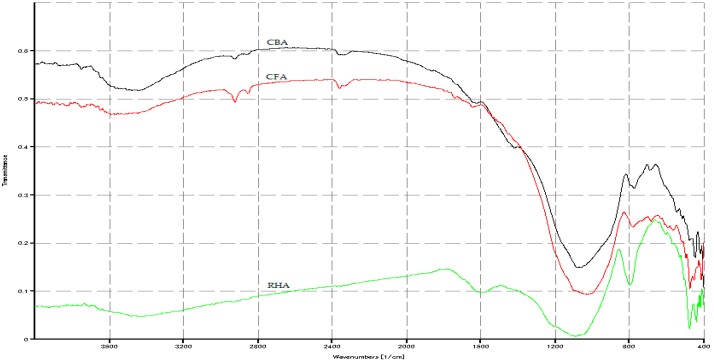
FTIR spectrograph of the raw materials.

The FTIR spectrographs in Figure 3 show H-O-H bond stretching and bending for the raw RHA at wavenumbers of 3435 cm^−1^ and 1595 cm^−1^ and CBA at a wavenumber of 3448 cm^−1^. These indicate that RHA and CBA have higher absorbed moisture as collected from their sources. All three materials, CFA, CBA and RHA, presented Si-O-Si, Si-O- bonds with RHA at 1101 cm^−1^, CBA at 1063 cm^−1^ and CFA at 1029 cm^−1^. Si-O, Si-O-Al bonds were seen at 780 cm^−1^ for CFA and at 774 cm^−1^ for CBA. Si-O-Fe bonds were seen at 475 cm^−1^ for CFA and 445 cm^−1^ for CBA.

**Figure 4:**
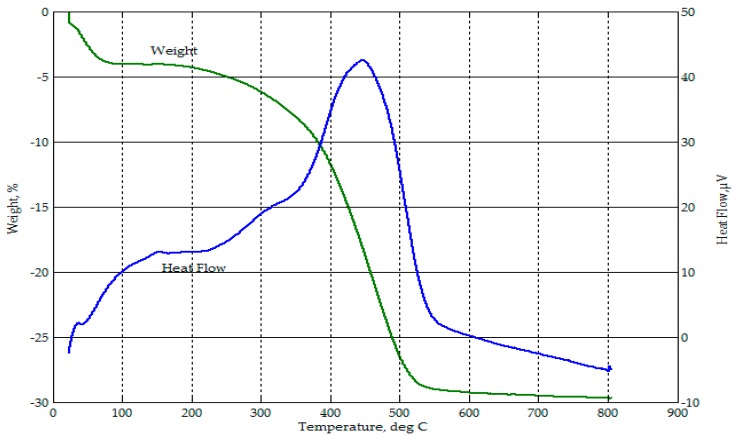
Thermograph of RHA.

Thermogravimetric analysis from ambient temperature to 800 °C on the RHA (Figure 4) showed a mass loss of 3.8% up to about 100 °C, attributed to moisture loss. This excess moisture was also seen from the FTIR spectrograph of RHA in Figure 3. From 300 °C to about 500 °C, there was an accelerating drop in mass to a total of 24.6%, which corresponded to an exothermic event. This may be attributed to the release of volatiles followed by oxidation processes due to combustibles (unburned carbon and rice hulls) in the sample. Beyond 500 °C, only a slight mass loss occurred. For this thermograph, the loss on ignition (LOI) of RHA was near 30%.

**Figure 5:**
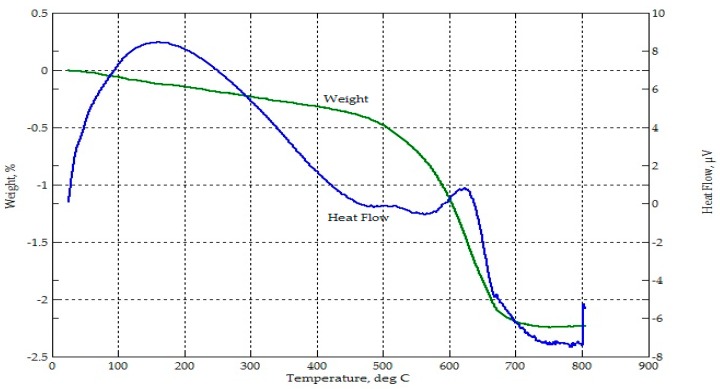
Thermograph of CFA.

**Figure 6:**
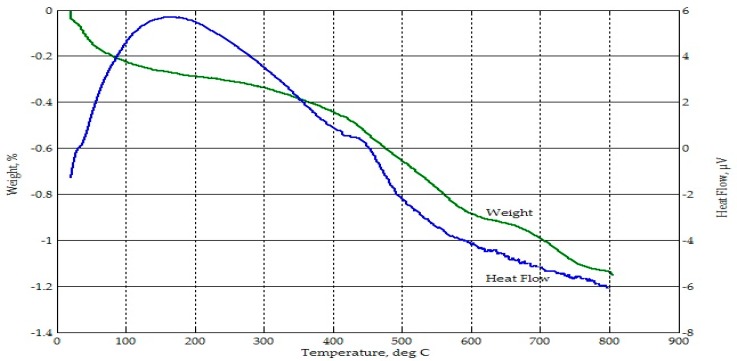
Thermograph of CBA.

From Figure 5 and Figure 6, thermogravimetric analyses of CFA and CBA indicated mass losses of less than 1% until 500 °C. This may be attributed to the release of volatile matter [51]. There was a higher rate of mass loss for CFA between 600 °C and 700 °C. Overall, both CFA and CBA were thermally stable within the temperature range considered. The loss on ignition (LOI) for CFA and CBA was a little over 2% and 1%, respectively.

**Figure 7:**
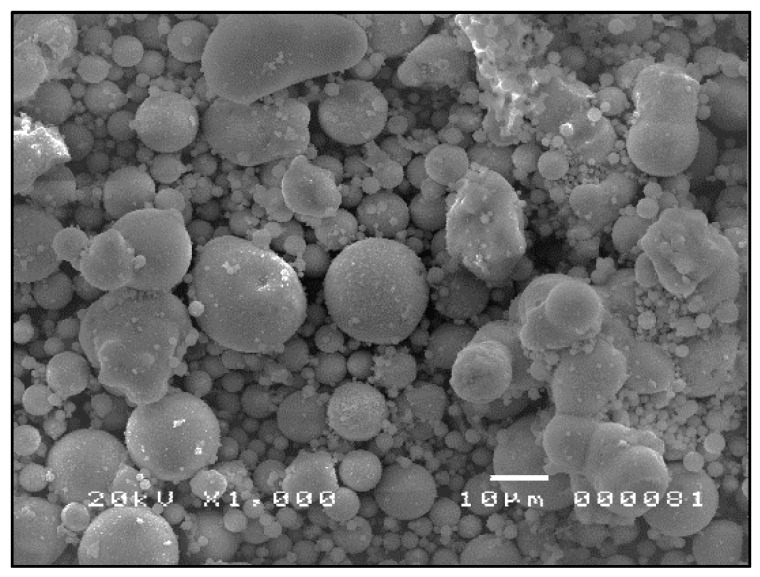
SEM micrograph of CFA ×1000.

**Figure 8:**
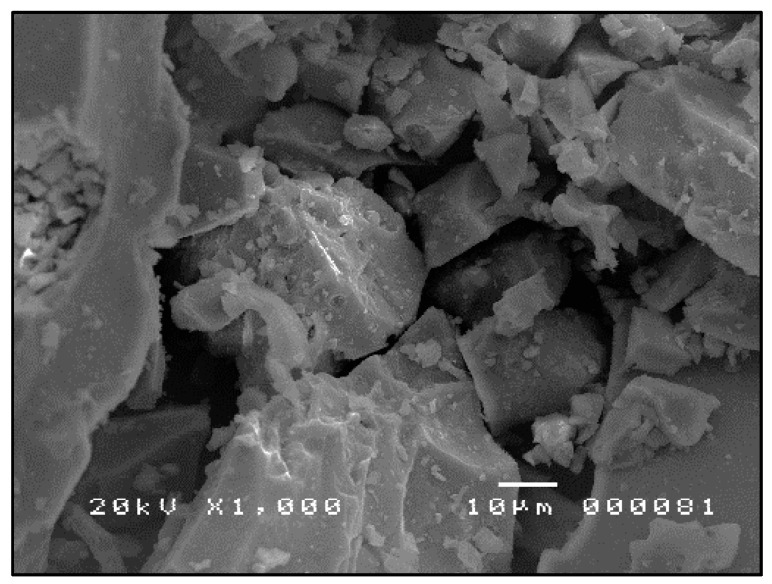
SEM micrograph of CBA (ground and sieved at 250 µm) ×1000.

**Figure 9:**
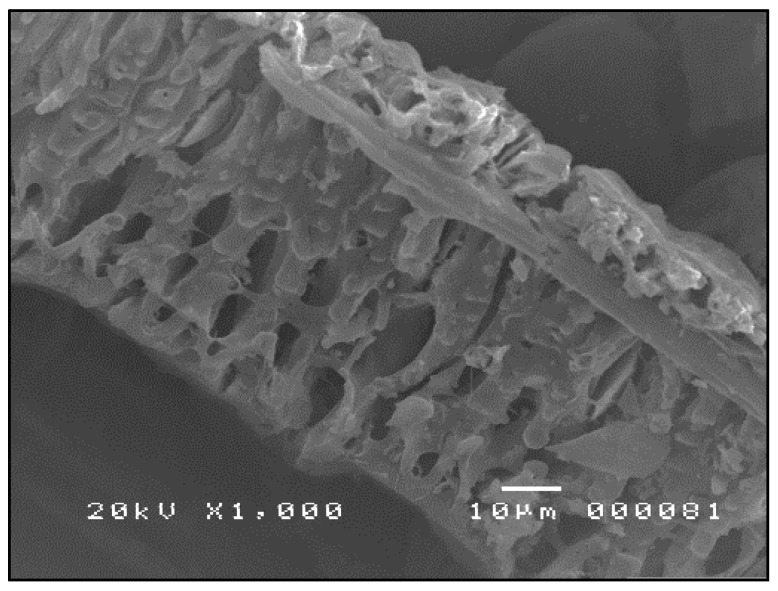
SEM micrograph of RHA ×1000.

The SEM micrographs shown in Figure 7, Figure 8 and Figure 9 are typical of CFA, CBA and RHA. The globules seen in Figure 7 for CFA are known to be alumino-silicate spheres and iron-rich spheres. XRD and XRF analyses of this CFA indicate the presence of these materials. CBA particles are irregular, but also visibly porous. The bigger sizes and void spaces as seen in the SEM micrograph of the RHA sample (Figure 9) also suggest the high porosity of the RHA raw material.

### 3.2. Optimal Mix Formulation for Ternary-Blended Geopolymer

#### 3.2.1. Mixture Design

Table 5 shows a summary of the engineering properties of geopolymer specimens prepared from the mix design of the raw materials. Based on the statistical analysis of these data, Equations (11)–(13) are the regression models for 28-day compressive strength (comp str), thermal conductivity (thr cond) and volumetric weight (vol wt) at the 5% level of significance. These were based on the backward elimination approach to determine the best-fitted model for each response variable as a function of the mix proportions of coal fly ash, coal bottom ash and rice hull ash. As shown in Table 6, the *p*-values are all <0.05, indicating the statistical significance of the regression models. In addition, the coefficients of determination (*R*-squared) are very near to 1.0, indicating that the data fit the model.

comp str = 13.1 × *F* + 12.6 × *B* + 1.22 × *R* + 2.58 × *F* × *B* − 8.33 × *F* × *R* − 9.39 × *B* × *R* − 115 × *F* × *B* × *R* (11)

thr cond = 0.480 × *F* + 0.790 × *B* + 0.230 × *R* − 0.700 × *F* × *B* − 0.330 × *F* × *R* − 1.05 × *B* × *R* + 5.96 × *F* × *B* × *R* (12)

vol wt = 1530 × *F* + 1650 × *B* + 570 × *R* (13)

where *F* = the mass fraction of CFA, *B* = the mass fraction of CBA and *R* = the mass fraction of RHA.

**Figure 10:**
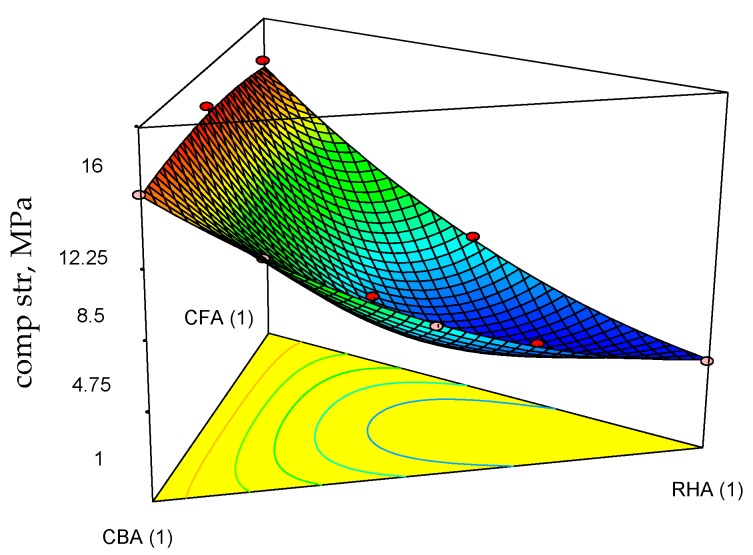
Response surface plots of the compressive strength of the geopolymer specimens and their projections onto the ternary diagram.

**Figure 11:**
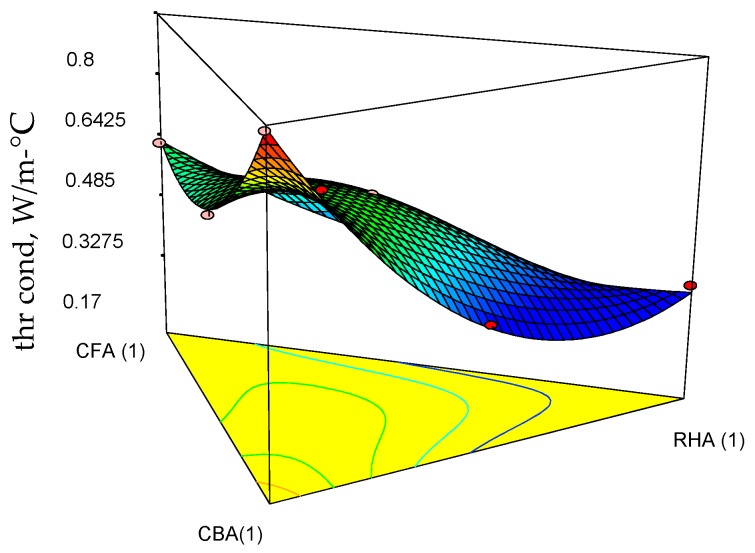
Response surface plots of the thermal conductivity of the geopolymer specimens and their projections onto the ternary diagram.

**Figure 12:**
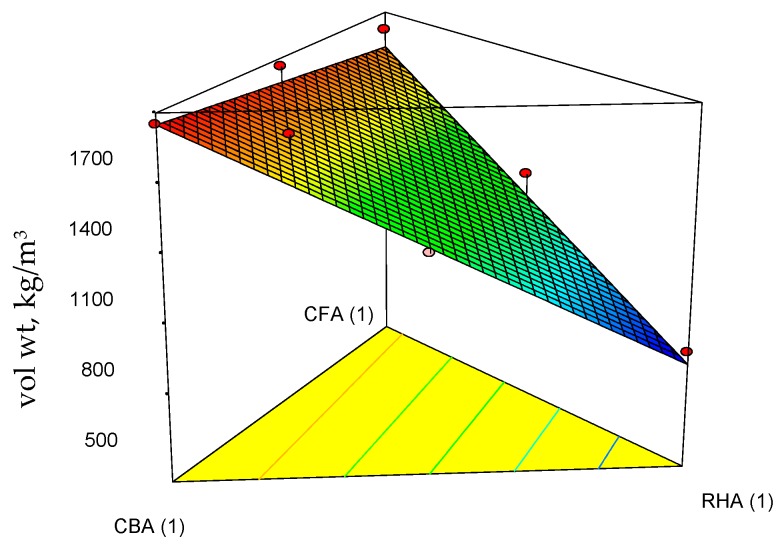
Response surface plots of the volumetric weight of the geopolymer specimens and their projections onto the ternary diagram.

The corresponding ternary plots for compressive strength, thermal conductivity and volumetric weights are shown in Figure 10, Figure 11 and Figure 12.

Focusing on the three features of the target applications, moderate strength (highest attained acceptable for moderately-loaded structures), low volumetric weight and low thermal conductivity, it is seen from Equation (11) and Figure 10 that CFA and CBA are the main contributors to increasing the compressive strength. Even the term involving the combination of CFA and CBA has a positive slope. However, all terms involving combinations of RHA with the other raw materials decrease the compressive strength. This effect of RHA to decrease the strength may be attributed to its significantly high LOI, as unburned carbon inhibits the process of geopolymerization [33]. It is seen from Equation (12) and Figure 11 that RHA contributes significantly to decreasing the thermal conductivity. Higher thermal conductivity leans towards a higher mass fraction of CBA. Additionally, it is seen from Equation (13) and Figure 12 that all components contribute linearly to the volumetric weight, but a higher proportion of RHA relative to coal ash would give lower volumetric weight if the desired product is a lightweight material.

#### 3.2.2. Multi-Response Surface Optimization

Given the multiple objective of (1) minimum volumetric weight, (2) minimum thermal conductivity and (3) compressive strength ≥11.7 MPa, with equal importance given to each objective, the optimum mix obtained was at 95.2-4.8 CFA-RHA mass ratio. The compressive strength was set to a minimum of 11.7 MPa to meet the performance standards for moderately-loaded OPC concrete as specified in ASTM C90-14 [42,43]. With a minimum of 15% CBA required, the optimum mix was at 85-15 CFA-CBA mass ratio. In consideration of the waste utilization of all three components and based on the mass fractions obtained in the first two models, with a minimum of 10% CBA and 5% RHA targeted, the recommended mix was at 85-10-5 CFA-CBA-RHA mass ratio. Table 7 summarizes these results together with the selected mixes for validation.

**Figure 13:**
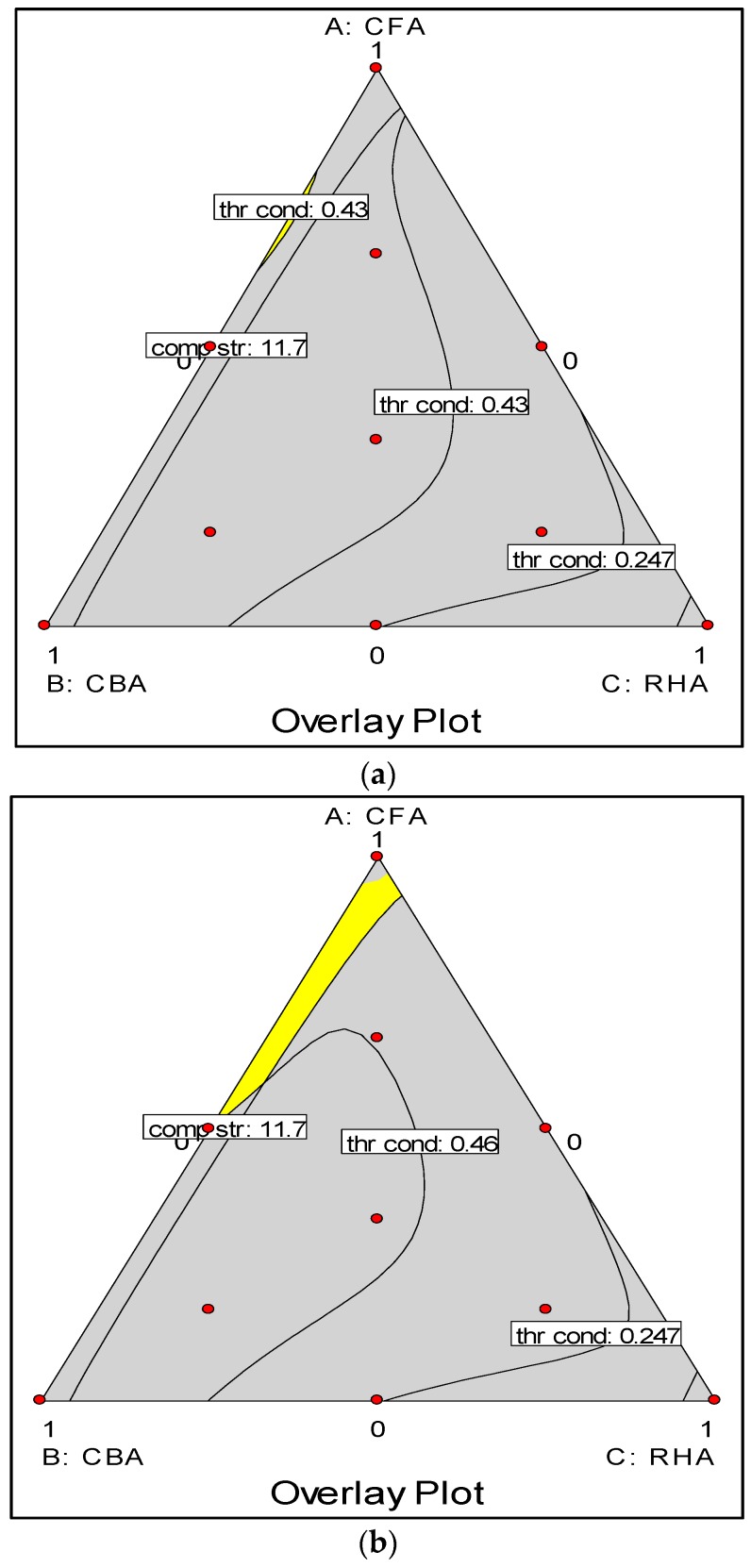

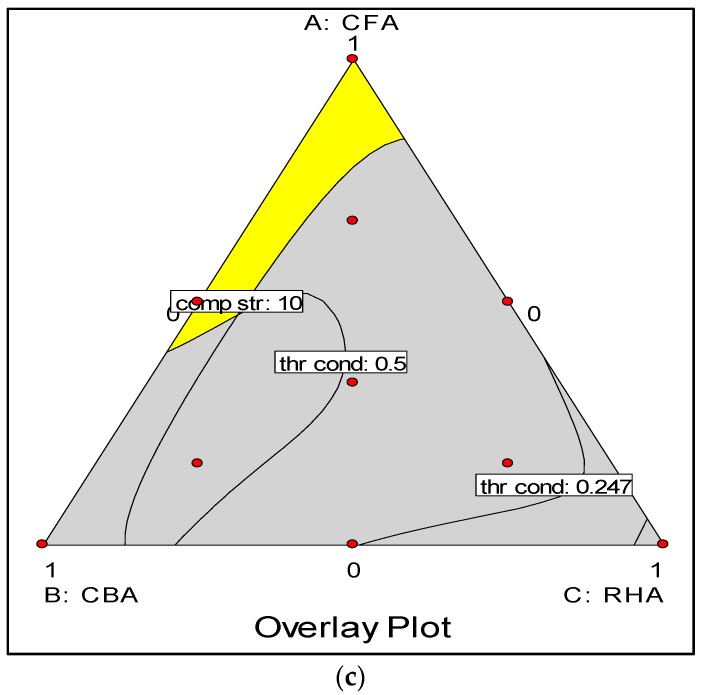
Overlay plots: (**a**) thermal conductivity <0.43 W/m-°C, compressive strength >11.7 MPa; (**b**) thermal conductivity <0.46 W/m-°C, compressive strength >11.7 MPa; (**c**) thermal conductivity <0.46 W/m-°C, compressive strength >10 MPa.

The overlay plots are shown in Figure 13. These plots were obtained subject to the criteria that: (1) the volumetric weight is below 1680 kg/m^3^ to conform with the performance specifications of lightweight concrete (ASTM C90-14) [42]; (2) the thermal conductivity is below 0.43 W/m-°C to be of comparable performance to insulating concrete (ASTM C332-09) [52]; and (3) compressive strength is above 11.7 MPa as specified for moderately loaded concrete (ASTM C90-14 and ASTM C0109/C0109M) [42,43]. The first criterion was met by all samples, thus only criteria (2) and (3) will determine the region of the solution. Figure 13a shows that these criteria were met within a very narrow band (shaded yellow) along the CFA-CBA edge (closer to CFA). If the thermal conductivity criterion is relaxed to below 0.46 W/m-°C, this band increases slightly, as seen in Figure 13b. If both the thermal conductivity (to below 0.46 W/m-°C) and compressive strength (to above 10 MPa) criteria are relaxed, this further increases the range of solutions as shown in Figure 13c. These plots indicate, as has been selected in Table 7, that the optimum mixes can be met with high CFA and much lower values of CBA and RHA.

**Figure 14:**
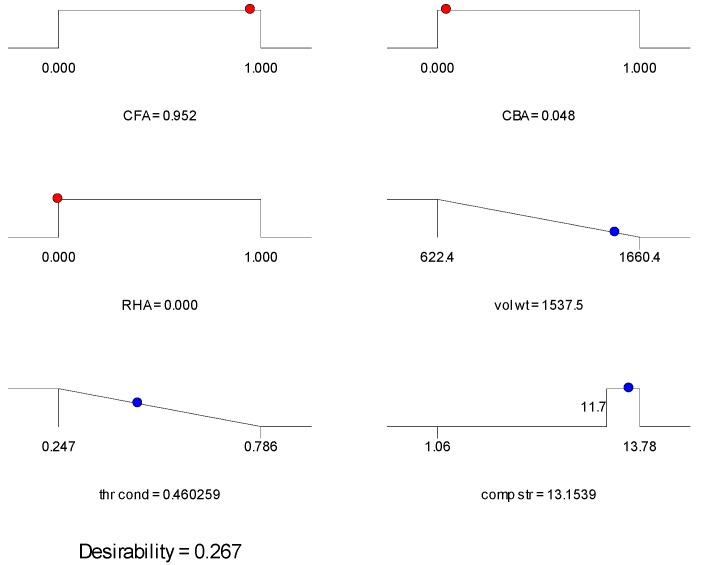
Ramp plot for Mix 1 of Table 7.

**Figure 15:**
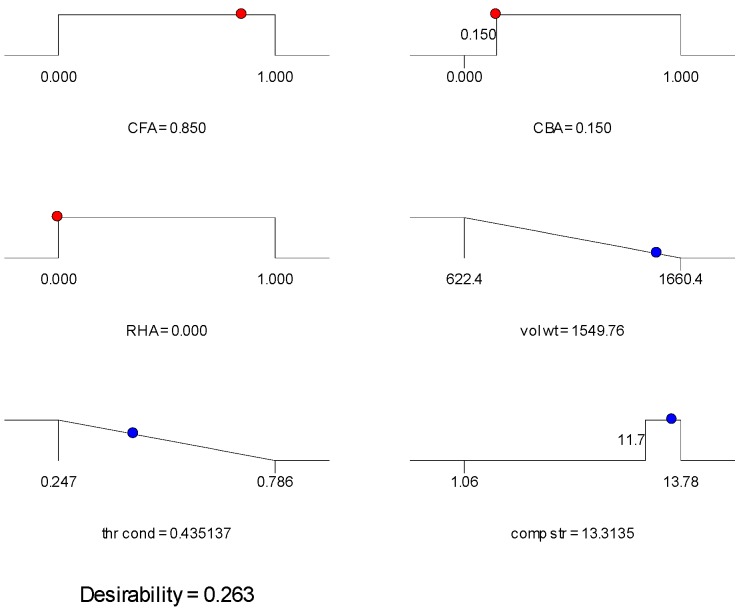
Ramp plot for Mix 2 of Table 7.

**Figure 16:**
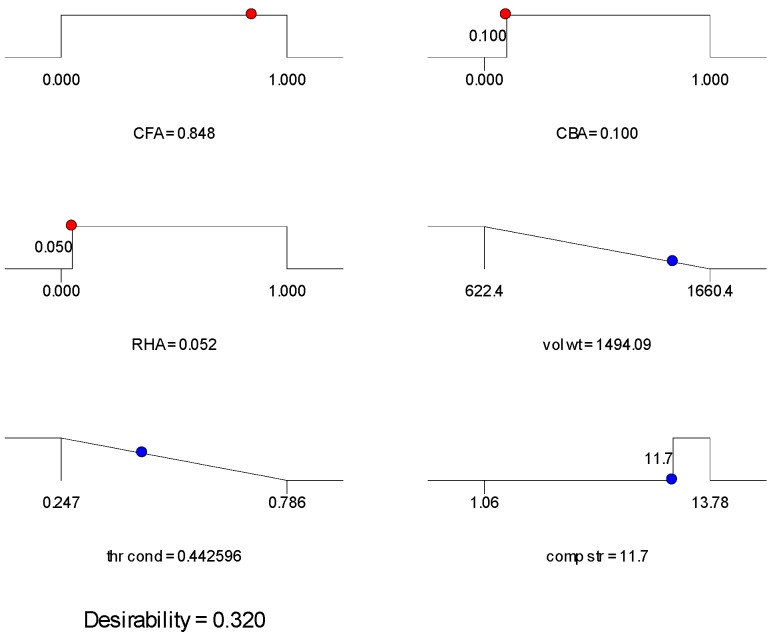
Ramp plot for Mix 3 of Table 7.

The ramp plots of the three mixes selected are shown in Figure 14, Figure 15 and Figure 16. The ramp plot indicates how the combined desirability of each mix is broken down to the individual desirability of each feature. In the ramp plots, the position of the dots along the horizontal indicates the values predicted for the solution having that mix ratio. The position of the dot along the vertical indicates the desirability. The higher the dot, the higher the desirability. For Mix 1 and Mix 2 (Figure 14 and Figure 15), compressive strength has high desirability, while volumetric weight and thermal conductivity have lower desirability. Mix 3 (Figure 16) has low individual desirability for all features, including compressive strength. The overall desirability for each mix is shown in Table 7.

Trace plots show the effect of the individual components on the response variables. The trace plots for Mix 1 are shown in Figure 17, Figure 18 and Figure 19, for Mix 2 in Figure 20, Figure 21 and Figure 22, and for Mix 3 in Figure 23, Figure 24 and Figure 25.

**Figure 17:**
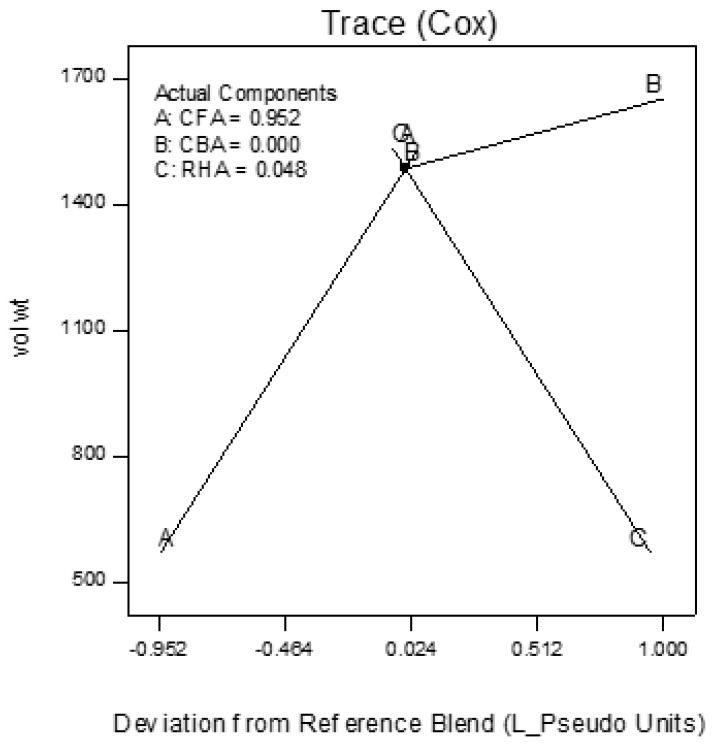
Trace plot for volumetric weight of Mix 1.

**Figure 18:**
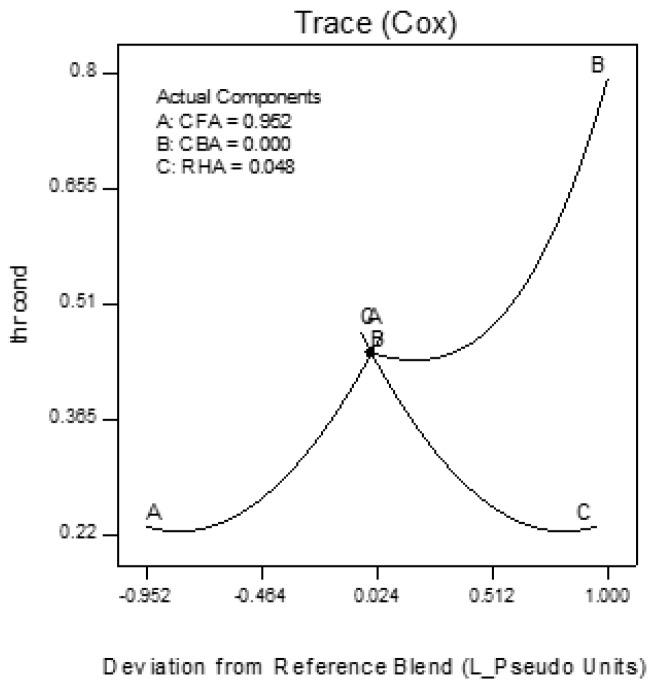
Trace plot for thermal conductivity of Mix 1.

**Figure 19:**
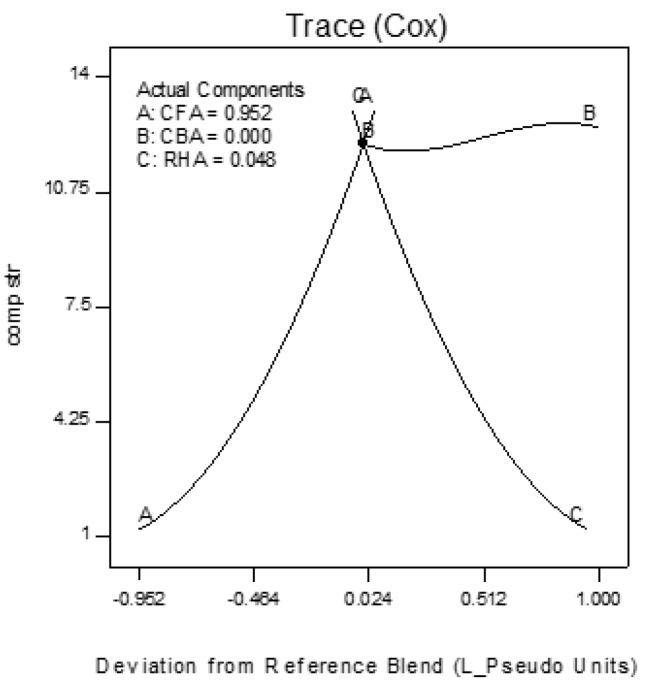
Trace plot for compressive strength of Mix 1.

For example, for Mix 1, Figure 17 shows that as CFA increases towards the mix ratio indicated, the volumetric weight increases, but as RHA increases, the volumetric weight decreases. Even as the reference value for CBA is zero, increasing from this value is expected to increase the volumetric weight. From Figure 18, the same behaviors for thermal conductivity are observed for all three components, but not linearly, as in volumetric weight (Figure 17). In Figure 19, it is seen that while compressive strength has the same behavior with CFA and RHA, it is not too sensitive to the deviation of CBA.

In all sets of trace plots, the following can be observed as the effect of blending CBA and RHA to CFA-based geopolymers: (1) increasing the RHA results in a significant decrease in the volumetric weight and thermal conductivity; however, there is also a significant decrease in compressive strength; and (2) volumetric weight and compressive strength are not too sensitive to deviations in CBA, but thermal conductivity increases with increasing CBA.

In comparison, as a mixing component for concrete, 7%, 10% and 15% by mass of RHA having 89.61% SiO_2_ and 5.91% LOI produced concrete with increasing compressive strengths as RHA was increased from 7%–15%. Compressive strength of as high as 46.9 MPa after 28 days was obtained at 15% RHA [53]. A study using rice hull bark ash (RHBA) [29], a mixture of rice hulls and eucalyptus bark with 81.36% SiO_2_ and 3.55% LOI, obtained the highest strength of about 34 MPa with 30% RHBA and using 12 M NaOH for geopolymers cured at 80 °C for 36 h and fully cured for 28 days. In a study that used CFA and CBA from circulating fluidized bed combustion (CFBC) [26], CFA was enhanced by an alkali-fusion pre-treatment at 350 °C for 0.5 h to increase its reactivity. The fused CFA was blended with ground CFBC bottom ash and activated by sodium silicate solutions. Geopolymer samples formed from this mixture were cured at 40 °C for seven days, reaching the highest compressive strength of 34.0 MPa. This suggests the importance of proper characterization and pre-treatment of raw materials to increase the reactivity of geopolymer precursors. Moreover, process conditions such as curing temperature for the production of geopolymer could also affect the engineering properties of the said materials.

From Table 7, the selected mixes for validation tests were: 95-5 CFA-RHA, 85-15 CFA-CBA and 85-10-5 CFA-CBA-RHA. An 85-15 CFA-CBA ratio was selected to include a binary mixture of CFA and CBA and since coal ash production in typical power plants was of the same ratio. The American Coal Ash Association reported in 2001 that both production and disposal of CFA and CBA were in the ratio of 80-20 [54]. The Japan Fly Ash Association in 2000 gave a ratio from an 85-15 to 95-5 mass ratio of CFA to CBA [55]. The selected ratio of 85-15 CFA-CBA then can potentially result in full utilization of all of the ash produced in a coal fired power plant.

### 3.3. Characterization of Geopolymers Formed

#### 3.3.1. FTIR of Geopolymers Formed

**Figure 26:**
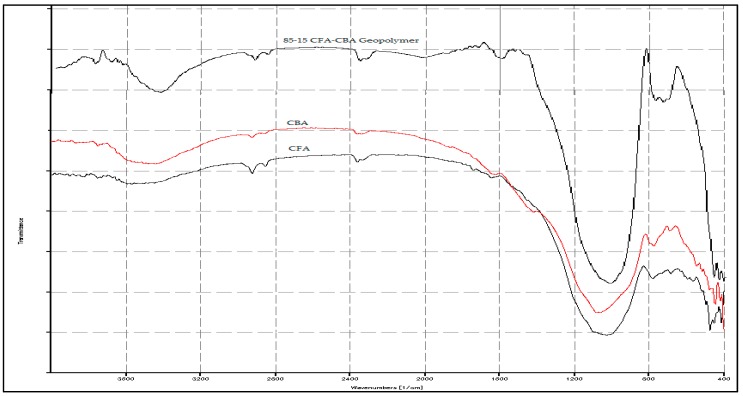
Spectrograph comparison of CFA, CBA raw materials and 85-15 CFA-CBA geopolymer.

From the FTIR spectrographs, as shown in Figure 26, the degree of geopolymerization with respect to each raw material can be determined from the ratio of peak heights at the Si-O-Si bonds [25]. For Figure 26, these are tabulated in Table 8.

As seen in Table 8, the peak ratio for CFA is higher than that for CBA. This indicates that the geopolymerization reactivity for CFA is higher than that of CBA. This supports the observation in the reactivity tests in Section 3.1 that the CFA used in this study has higher reactivity than the CBA used. It should be noted that in [25], the same observations were obtained. Furthermore, the shifting of the wavenumber of the Si-O-Si stretching to the right (to a lower wavenumber) is indicative of the breaking of Si-O bonds and the formation of new Si bonds in the process of geopolymerization.

**Figure 27:**
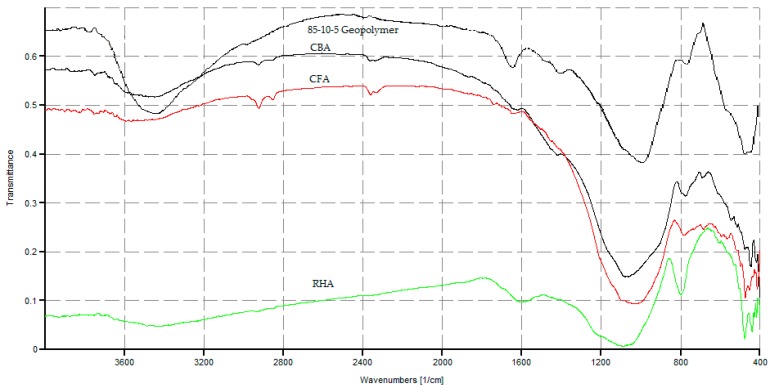
Comparison between the spectrographs of the CFA, CBA and RHA raw materials and the 85-10-5 FA-CBA-RHA geopolymer.

**Figure 28:**
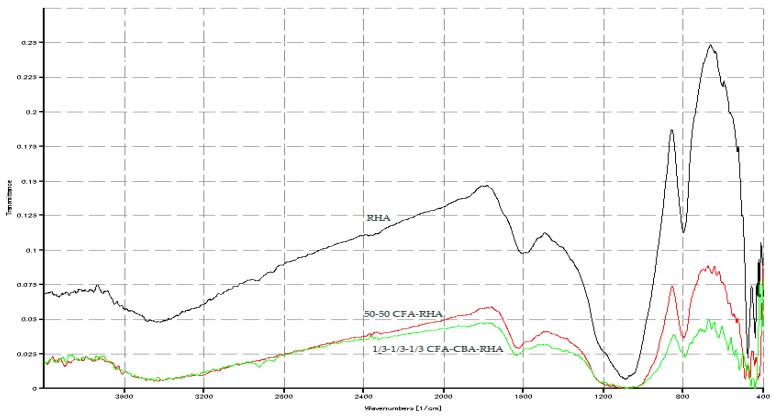
Spectrograph comparison of RHA raw material, 50-50 CFA-RHA geopolymer, and 1/3-1/3-1/3 CFA-CBA-RHA geopolymer.

In Figure 27, there is a significant shift of wavenumber to the right of the Si-O-Si bond for the 85-10-5 CFA-CBA-RHA geopolymer as compared to the starting raw materials. This indicates the high level of geopolymerization achieved in this mixture. However, in Figure 28, using the same indicator, geopolymers with a high amount of RHA (≥1/3) have very low levels of geopolymerization.

#### 3.3.2. SEM Analysis of the Geopolymers Formed

**Figure 29:**
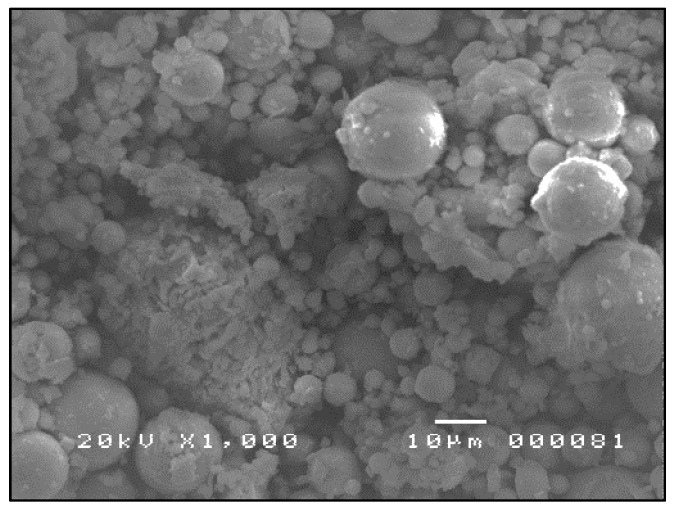
SEM micrograph of 95-5 CFA-RHA geopolymer ×1000.

**Figure 30:**
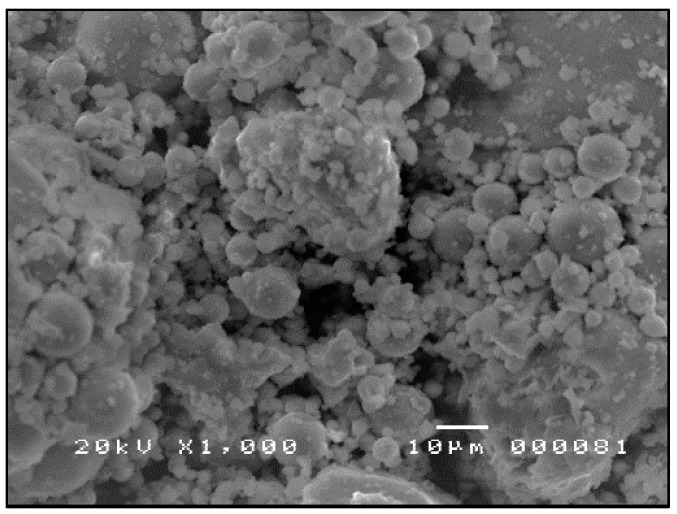
SEM micrograph of 85-15 CFA-CBA geopolymer ×1000.

**Figure 31:**
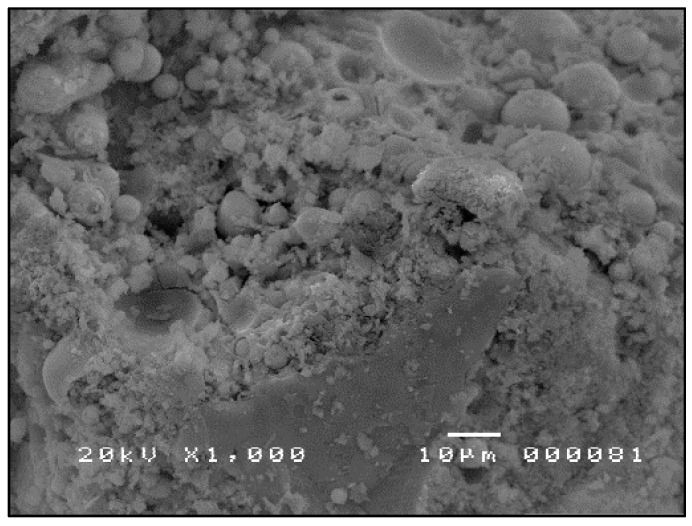
SEM micrograph of 85-10-5 CFA-CBA-RHA geopolymer ×1000.

Figure 29, Figure 30 and Figure 31 show the SEM micrographs of 95-5 CFA-RHA, 85-15 CFA-CBA and 85-10-5 CFA-CBA-RHA geopolymers. The 85-10-5 CFA-CBA-RHA geopolymer has the finest contours and surfaces indicative of the extent of dissolution and polycondensation that occurred during geopolymerization for this mixture.

#### 3.3.3. Thermogravimetric Analysis of the Geopolymers Formed

**Figure 32:**
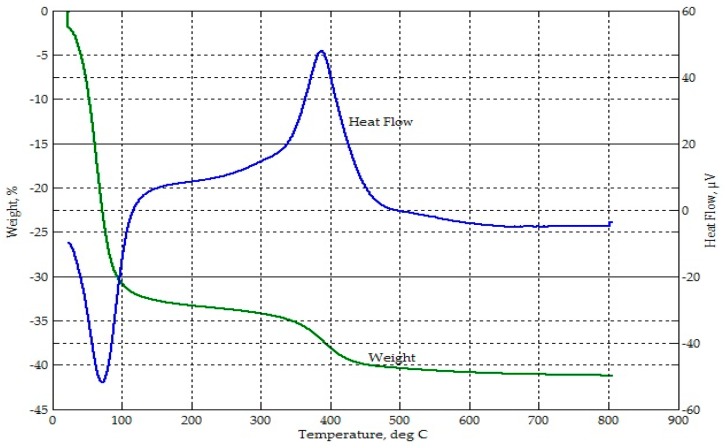
Thermograph of the 50-50 CFA-RHA geopolymer.

**Figure 33:**
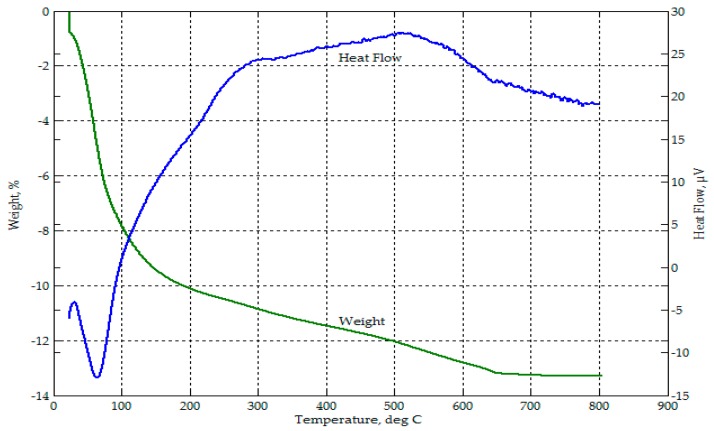
Thermograph of the 50-50 CFA-CBA geopolymer.

In the thermograph of 50-50 CFA-RHA, as shown in Figure 32, the mass loss from ambient temperature to about 110 °C was more than 30%. This mass loss can be attributed to water loss via evaporation. Water was present in the slurry mix as part of the alkali activator solution and as additional water added to improve workability. Some of this water were retained in the geopolymer specimens formed even after curing. In the thermograph of 50-50 CFA-CBA, as shown in Figure 33, the mass loss from ambient temperature to about 110 °C was only about 8%. In Figure 32, about 5% additional mass loss occurred in the 50-50 CFA-RHA geopolymer between temperatures of 350 °C and 450 °C. This can be attributed to the combustion of unburned carbon and rice hulls in the RHA. This occurrence was also observed in the thermograph of RHA in Figure 4. This event was not observed in the thermograph of the 50-50 CFA-CBA geopolymer (Figure 33). However, besides the drying process that occurred from ambient temperature to 350 °C, the 50-50 CFA-RHA geopolymer can be considered as thermally stable within the temperature range considered. The 50-50 CFA-CBA has no significant event and, thus, can be considered as thermally stable throughout the range of temperatures used.

### 3.4. Effect of Pre-Curing Temperatures on Optimized Geopolymer Mix

**Figure 34:**
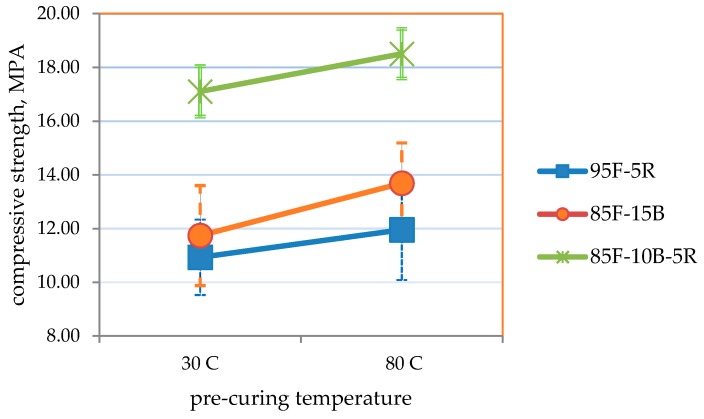
Compressive strength of the optimum mixes selected vs. 24-h pre-curing temperature.

**Figure 35:**
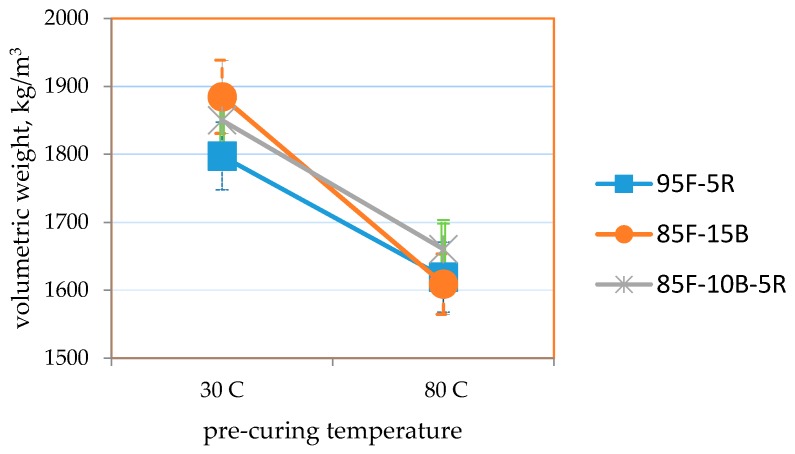
Volumetric weight of the optimum mixes selected vs. 24-h pre-curing temperature.

**Figure 36:**
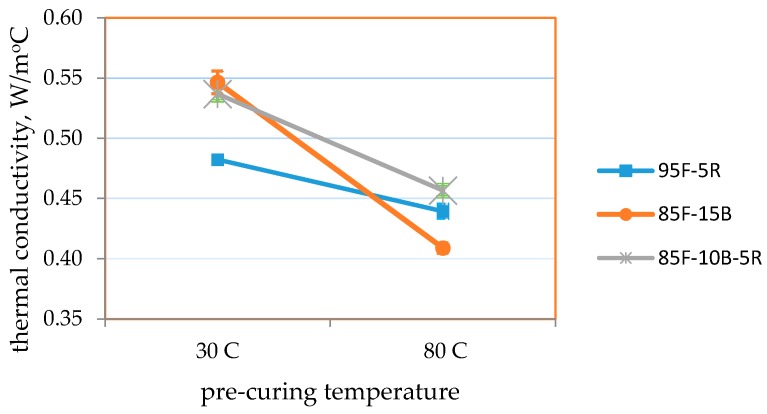
Thermal conductivity of the optimum mixes vs. 24-h pre-curing temperature.

Table 9 and Figure 34, Figure 35 and Figure 36 summarize the results of the effect of pre-curing temperature. Results for samples cured at ambient conditions (30 °C) shows that the strength and thermal conductivity values are within the target values, but the volumetric weight is higher than the target but not more than 1680 kg/m^3^. This could be attributed to the more compact formation of samples and the longer time allotted for the removal of air bubbles via manual vibration.

It is seen that samples pre-cured at 80 °C attained higher strengths, lower volumetric weight and lower thermal conductivity for all mixes. The direct relation between lower volumetric weight and lower thermal conductivity also reveals that moisture content directly affects thermal conductivity. Even as initial moisture content may be higher at the start of curing, removal of moisture through drying will create void spaces within the geopolymer block [56], which results in lower thermal conductivity [57]. Thermal conductivity also directly correlates with moisture content [57]. This may be attributed to the higher thermal conductivity of water from 30 °C to 80 °C (0.615 W/m-°C to 0.67 W/m-°C) [58] compared to the dry geopolymer.

As specified in ASTM C90-14 and ASTM C109/C109M-02 [42,43], moderately-loaded, lightweight concrete should have a compressive strength ≥11.7 MPa and a volumetric weight ≤1680 kg/m^3^. ASTM C332-09 [52] specifies thermal conductivity ≤0.43 W/m-°C for lightweight insulating concrete.

Results show that the highest compressive strength (18.5 MPa) was obtained for an 85-10-5 CFA-CBA-RHA geopolymer pre-cured at 80 °C. For this mix, the volumetric weight is 1660 kg/m^3^ and the thermal conductivity is 0.457 W/m-°C. The lowest volumetric weight at 1610 kg/m^3^ and the lowest thermal conductivity of 0.410 W/m-°C were obtained from 85-15 CFA-CBA geopolymer pre-cured at 80 °C. For this mix, the compressive strength is 13.7 MPa. All of these values for this mix satisfy the requirements for the target applications.

### 3.5. Embodied Energy and Embodied CO_2_ of Optimized Geopolymer Mix

Embodied energy and embodied CO_2_ were estimated for the optimal mixes selected. Assigning the embodied impacts of the ashes to the products delivered in their generation (i.e., to electricity when coal or rice hulls are used as fuels in power plants), the ashes come in with negligible embodied impacts [47]. The reference values of embodied energy and embodied CO_2_ for the ashes and the alkali activator components are given in Table 10. The estimated values of embodied energy and embodied CO_2_ due to the transport of raw materials are presented in Table 11. Embodied energy and embodied CO_2_ associated with materials preparation and geopolymerization process are not yet included. These processes include drying, grinding and sieving of dry materials and pre-curing at elevated temperature of the geopolymers formed. These may be assumed comparable to that obtained in the production of OPC [47]. However, the study emphasizes minimal raw materials processing with only CBA ground and passed through a 250-μm sieve and the drying and pre-curing processes expected to be fueled via rice hull combustion. Compared to OPC, which is processed at much higher temperatures, the inclusion of these materials processing impacts in the study will favor geopolymers with much smaller additional embodied energy. This will increase the embodied CO_2_ due to the additional transportation of rice hulls. However, the combustion of rice hulls will offset the hauling of RHA and, thus, counterbalance the additional transport effects.

The corresponding embodied energy and embodied CO_2_ equations are:

Embodied energy = 0.187 × *F* + 0.237 × *B* + 0.111 × *R* + 1.50 MJ/kg (14)

CO_2_/kg = 0.0258 × *F* + 0.0308 × *B* + 0.0169 × *R* + 0.092 kg CO_2_/kg (15)

where *F* = the mass fraction of CFA, *B* = the mass fraction of CBA and *R* = the mass fraction of RHA.

From Table 12, it is seen that the embodied energy obtained is at 1.64 MJ/kg, and the embodied CO_2_ ranged from 0.117–0.118 kg CO_2_/kg. The embodied energy of the three mixes is the same since the mass ratio of alkali solution to dry materials used is the same and the amount of water added is also maintained at the same amount. The embodied CO_2_ varies slightly as the mix proportions of the ashes vary.

It should be noted that the computed embodied energy of 1.64 MJ/kg and embodied CO_2_ of 0.12 kg CO_2_/kg for this optimized geopolymer mix are lower than that of Portland cement-based materials. For example, for pure OPC, the embodied energy and embodied CO_2_ are 4.6 MJ/kg and 0.83 kg CO_2_/kg, respectively. At 50% replacement with fly ash in blended OPC, the embodied energy and embodied CO_2_ are 2.43 MJ/kg and 0.42 kg CO_2_/kg, respectively [44].

## 4. Conclusions

The fundamental idea behind all of these investigations and studies on geopolymer characteristics and applications is that it is possible to obtain the best performance for any suitable precursor materials used in the geopolymerization process via materials processing, blending and activation procedures. Conversely, it is also a strategy to limit materials processing to the most practicable and still meet the requirements of a target application. In this study, with the further goal of including all three waste materials, a ternary mixture of 85-10-5 by mass ratio of CFA-CBA-RHA was developed that was able to meet the requirements of the target application. The ratio of CFA-CBA approximates the typical ash generation ratio in modern coal-fired power plants, which may be envisioned as a possibility for full coal ash utilization. The addition of RHA engages us to extend the exploration of sustainability to complete the energy and material cycle via rice hull combustion and production of RHA to feed the cycle for producing “green” materials. However, it should be understood also that materials and processes, including geopolymerization, may be better implemented by matching to appropriate applications. The results of this study may be considered as one particular case.

The properties of geopolymer-based materials are dependent on the blending components and processing conditions used; thus, the results obtained are for the specific local materials and processing conditions used in this study. As the raw materials used have characteristic limitations (i.e., the high LOI of the RHA) and the minimal processing done (i.e., the size of the CBA being reduced to only 250 µm), producing the target results is an endorsement of this investigation and an invitation for future research.

## Figures and Tables

**Figure 20:**
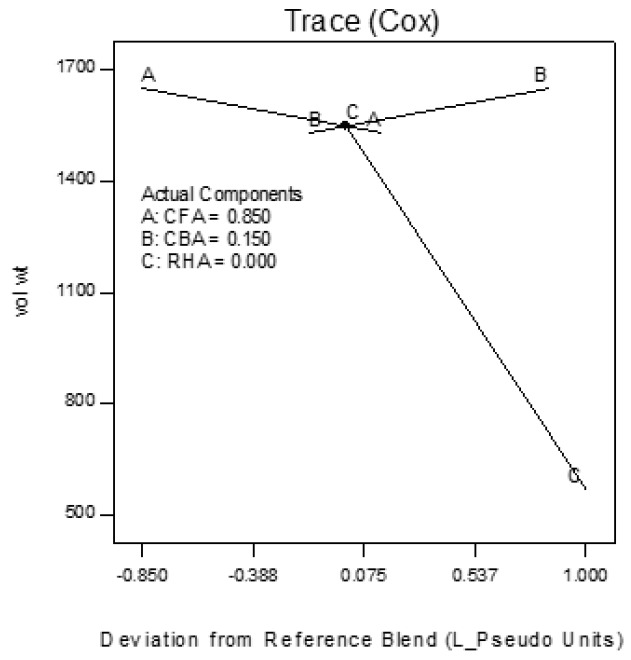
Trace plot for volumetric weight of Mix 2.

**Figure 21:**
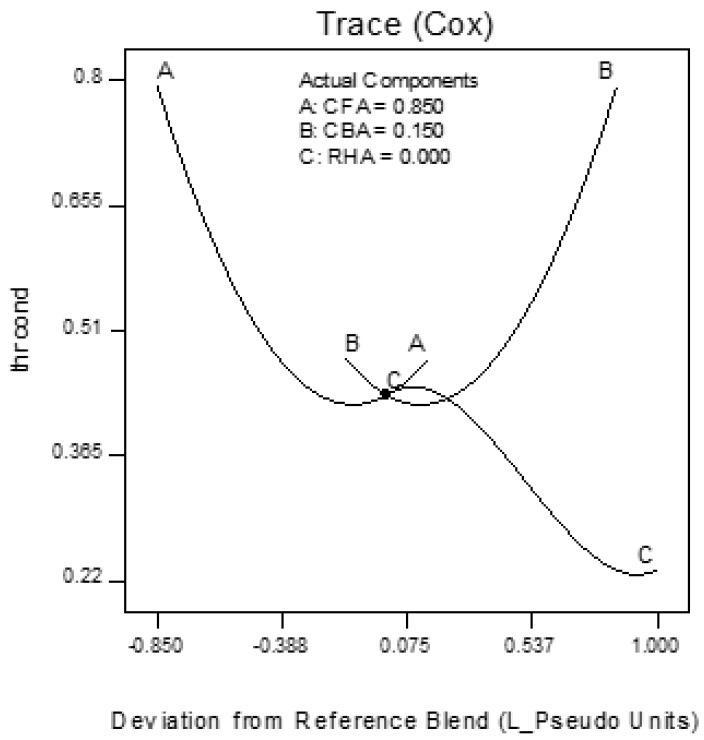
Trace plot for thermal conductivity of Mix 2.

**Figure 22:**
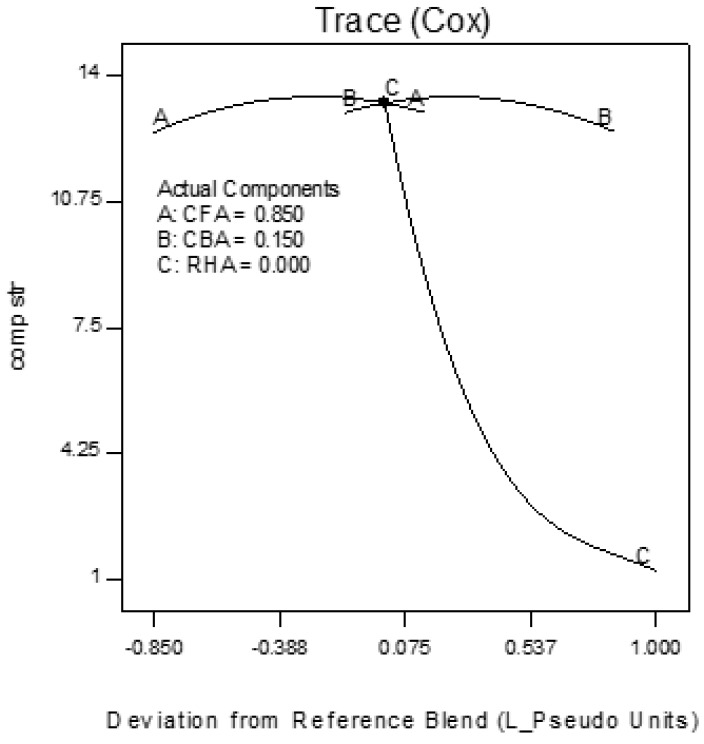
Trace plot for compressive strength of Mix 2.

**Figure 23:**
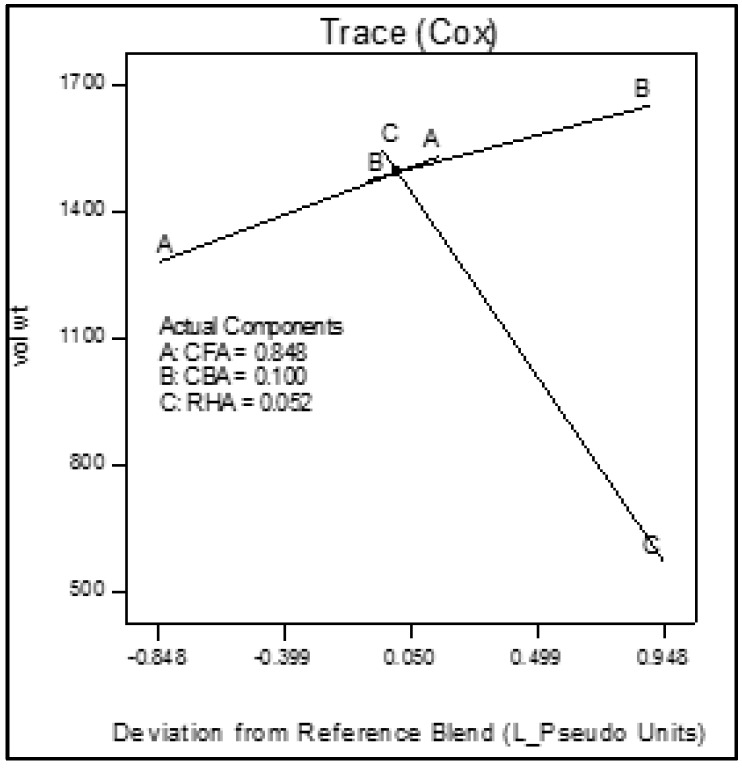
Trace plot for volumetric weight of Mix 3.

**Figure 24:**
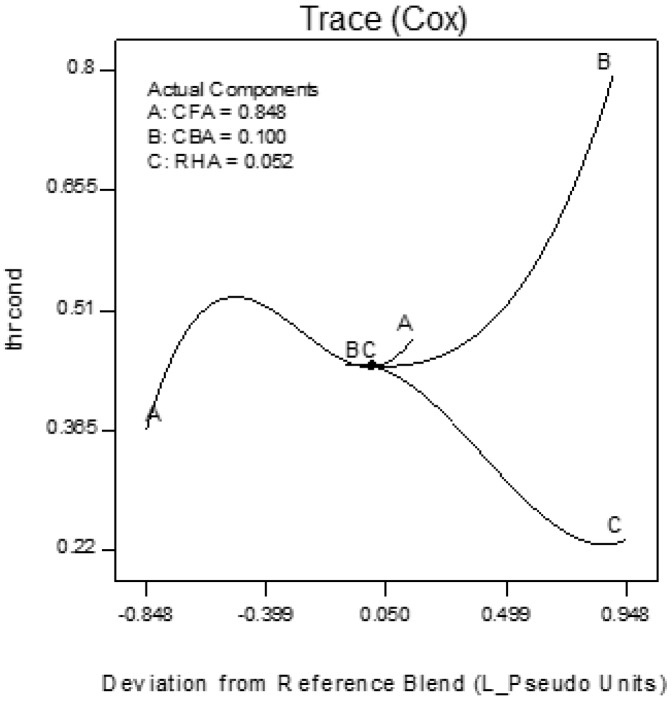
Trace plot for thermal conductivity of Mix 3.

**Figure 25:**
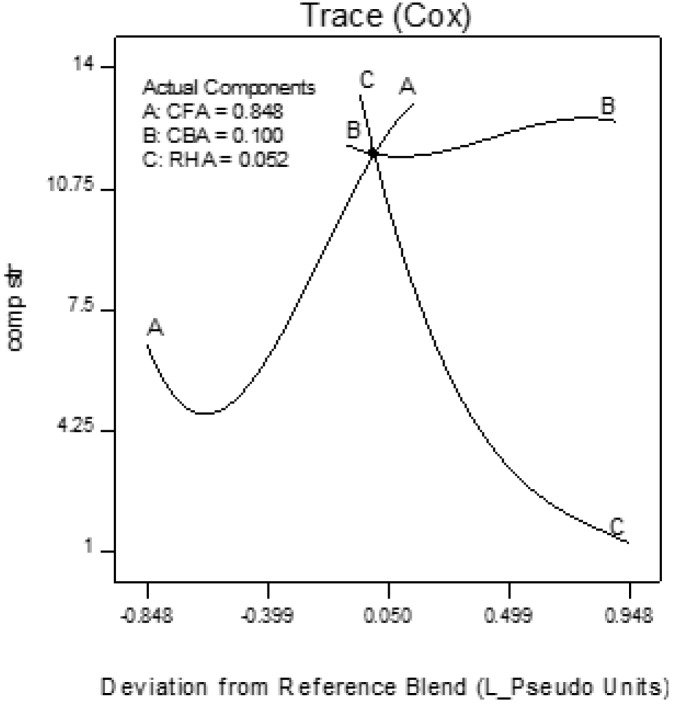
Trace plot for compressive strength of Mix 3.

**Table 1:** Mix design expressed as the mass fraction of raw materials.

Mix Ratio	CFA	CBA	RHA
A1	1	0	0
A2	0	0	1
A3	0	1	0
A4	0.500	0	0.500
A5	0	0.500	0.500
A6	0.500	0.500	0
A7	0.167	0.167	0.666
A8	0.666	0.167	0.167
A9	0.167	0.666	0.167
A10	0.333	0.333	0.333

**Table 2:** Composition of raw materials using XRF (as mass percentage) and the molar ratios of oxides. LOI, loss on ignition.

Raw Material	Al_2_O_3_	SiO_2_	Cl	K_2_O	CaO	TiO_2_	Cr_2_O_3_	Fe_2_O_3_	LOI	K_2_O/Al_2_O_3_	K_2_O/SiO_2_	SiO_2_/Al_2_O_3_
CFA	21.8	66.5		1.49	5.30	0.40		2.52	2.18	0.074	0.014	5.20
CBA	18.4	57.0	0.76		11.1	1.14	0.08	10.5	1.07			5.24
RHA		70.1		1.10	0.19				28.6		0.01	

**Table 3:** Dissolution tests on raw materials.

Raw Material	NaOH, mL	Initial Mass, g	Dissolved, g	% Dissolved
CFA	50.0	2.51	0.770	30.7
CBA	50.0	2.54	0.359	14.1
RHA	50.0	2.09	0.502	24.0

**Table 4:** ICP analysis results on 10 M NaOH solvent used in the dissolution tests (values are in ppm).

Raw Material	K	Si	Al	Fe	Zn	Sr
RHA	690	2260	4.28	0.020	0.500	0.010
CBA	67.5	188	19.2	2.12	0.600	0.150
CFA	63.4	224	127	13.1	0.920	0.050

**Table 5:** Mixture ratios and engineering properties measured from the geopolymers formed.

Mix Ratio	CFA	CBA	RHA	vol wt	thr cond	comp str
A1	1	0	0	1610	0.469	13.4
A2	0	0	1	622	0.247	1.06
A3	0	1	0	1650	0.786	12.6
A4	0.500	0	0.500	1150	0.277	5.23
A5	0	0.500	0.500	1090	0.258	4.38
A6	0.500	0.500	0	1660	0.444	13.8
A7	0.167	0.167	0.666	844	0.262	1.38
A8	0.666	0.167	0.167	1180	0.481	6.82
A9	0.167	0.666	0.167	1520	0.580	7.48
A10	0.333	0.333	0.333	1180	0.486	3.49

Legend: vol wt = volumetric weight, kg/m^3^; thr cond = thermal conductivity, W/m-°C; comp str = compressive strength, MPa.

**Table 6:** ANOVA for the significance of the regression models at α = 0.05 using backward elimination.

Model	*F*-Ratio	*p*-Value	Mean	Std. Dev.	*R*-Squared
Equation (11)	51.5	0.0041	6.96	0.810	0.990
Equation (12)	35.3	0.0071	0.43	0.036	0.986
Equation (13)	43.7	0.0001	1250	110	0.926

**Table 7:** Summary of the optimal mixes derived from multi-response optimization via the desirability function.

Mix	Constraints	CFA	CBA	RHA	vol wt	thr cond	comp str	Desirability	Selected Mix
1	Min vol wt Min thr cond Comp str ≥ 11.7	0.952	0.000	0.048	1540	0.460	13.2	0.267	95-5 CFA-RHA
2	CBA ≥ 15% Min vol wt Min thr cond Comp str ≥ 11.7	0.850	0.150	0.000	1550	0.435	13.3	0.263	85-15 CFA-CBA
3	CBA ≥ 10% RHA ≥ 5% Comp str ≥ 11.7	0.848	0.100	0.052	1490	0.443	11.7	0.320	85-10-5 CFA-CBA-RHA

**Table 8:** Peak height ratios for Si-O-Si bond stretching.

Sample	Wavenumber, cm^−1^	Peak Ratio
85-15-cfa-cba	1030	
CBA raw	1079	2.60
CFA raw	1047	3.98

**Table 9:** Measured properties of the optimum mixes selected at 30 °C and 80 °C 24-h pre-curing temperature. Corresponding charts are in Figure 34, Figure 35 and Figure 36.

Samples	Temperature	Volumetric Weight, kg/m^3^	Thermal Conductivity, W/m-°C	Compressive Strength, MPa
95-5-CFA-RHA	30 °C	1800 ± 50	0.482 ± 0.010	10.9 ± 1.4
	80 °C	1620 ± 52	0.439 ± 0.006	12.0 ± 1.9
85-15-CFA-CBA	30 °C	1890 ± 54	0.547 ± 0.009	11.7 ± 1.9
	80 °C	1610 ± 45	0.409 ± 0.004	13.7 ± 1.5
85-10-5-CFA-CBA-RHA	30 °C	1850 ± 36	0.537 ± 0.006	17.1 ± 0.9
	80 °C	1660 ± 40	0.457 ± 0.005	18.5 ± 0.9

**Table 10:** Reference values of embodied energy for the materials used in the study.

Material	Embodied Energy, MJ/kg	kg CO_2_/kg	Source/Reference [44,58]
CFA	0.10	0.010	Hammond and Jones, 2011
CBA	0.15	0.015	Hammond and Jones, 2011
RHA	0.10	0.015	Hammond and Jones, 2011
Water	0.01	0	Hammond and Jones, 2011
waterglass	16	1.35	Maskell et al., 2014
Sodium hydroxide	23	1.30	Maskell et al., 2014

**Table 11:** Estimated values of embodied energy for the transport of materials used in the study.

Raw Materials	Distance Travelled, km	Liters Diesel Consumed	kg Per Truck	Embodied Energy *, MJ/kg	Embodied CO_2_ *, CO_2_/kg
CFA	124	12.4	5000	0.0866	0.0158
CBA	124	12.4	5000	0.0866	0.0158
RHA	15.3	1.53	5000	0.0107	0.0019

* Computed on the basis of 10 km/L of diesel, energy value of 34.92 MJ/L of diesel and 5 MT/truckload.

**Table 12:** Computed embodied energy and embodied CO_2_ for the mixes selected.

Mix Ratio	CFA *	CBA *	RHA *	NaOH *	WGS *	Water *	Embodied Energy, MJ/kg	Embodied CO_2_, kg CO_2_/kg
1	0.95	0	0.05	0.052	0.018	0.180	1.64	0.117
2	0.85	0.15	0	0.052	0.018	0.180	1.64	0.118
3	0.85	0.10	5	0.052	0.018	0.180	1.64	0.118

* The values are mass fractions with respect to dry materials (CFA + CBA + RHA = 1.0).

## Abbreviations

The following abbreviations are used in this manuscript:

MMT	million metric tons
CFA	coal fly ash, also F as used in the equations
CBA	coal bottom ash, also B as used in the equations
RHA	rice hull ash, also R as used in the equations
XRF	X-ray fluorescence
XRD	X-ray diffraction
ICP	inductively-coupled plasma mass spectrometry
FTIR	Fourier transform infrared spectroscopy
SEM	scanning electron microscope
TGA-DTA	thermogravimetric analysis-differential thermal analysis
HVFA	high volume fly ash concrete
SCM	supplementary cementitious materials
OPC	ordinary Portland cement
